# Metabolic Control of B Cells: More Questions than Answers

**DOI:** 10.3389/fimmu.2015.00080

**Published:** 2015-02-25

**Authors:** Melania Capasso, Alaa Rashed Alyahyawi, Sarah Spear

**Affiliations:** ^1^Centre for Cancer and Inflammation, Barts Cancer Institute, Queen Mary University of London, London, UK

**Keywords:** B lymphocytes, obesity and inflammation, insulin resistance, B regulatory cells, inflammatory cytokines

It is now accepted that metabolic disorders such as type 2 diabetes (T2D) and obesity are characterized by alterations in immune cells. Indeed, immune cells have been shown to play a critical role in fueling the inflammation that accompanies increased fat deposition and insulin resistance. Both the innate (macrophages) and the adaptive arm (CD4 and CD8 T cells, in particular) significantly contribute to the chronic inflammatory state that characterizes these conditions. B cells, however, have until recently remained significantly understudied. This is not due to the fact that they do not play as significant a role, as more recent publications appear to show. Here, we briefly review the most recent literature and highlight the remaining outstanding questions.

## A Cross Talk between B Cells and Adipocytes?

B lymphocytes or B cells are the arm of the adaptive immune system responsible for the generation of antibodies. In addition, they produce cytokines that support the activation of other immune cells (1). Whether they interact and influence the physiology or pathophysiology of other non-immune cells, such as adipocytes in obesity, is only now beginning to emerge.

Adipocytes themselves secrete cytokines also secreted by immune cells, such as TNFα, IL-6, and IL-8 (2). However, adipocytes secrete cytokines at significantly lower levels than the immune cells infiltrating adipose tissue in obesity (2). Adipocytes also secrete adipocyte-specific cytokines known as adipokines, these include leptin, adiponectin, resistin, which are also altered in obesity (3). The receptors for both leptin and adiponectin have been found on B cells; however, their role is still not understood. There is some evidence that leptin induces secretion of IL-10, IL-6, and TNFα in human B cells (4). However, how adipokines affect B cells and whether this plays a role in obesity has not been sufficiently addressed yet.

## B Cells in Obese Adipose Tissue and T2D: Similarities and Differences between Mouse and Man

Recently, a number of studies have revealed B cells contribute to the progression and severity of obesity and T2D.

During weight gain, the earliest infiltrators into adipose tissue are IFNγ-secreting CD8+ T cells, then followed by M1-polarized, inflammatory macrophages, which secrete IL-6, IL-12, and TNFα (5). Conversely, anti-inflammatory mediators such as CD4+ T regulatory cells and M2-polarized macrophages are reduced in obese adipose tissue (6). The activation of pro-inflammatory immune cells during obesity is likely caused by the release of free fatty acids (FFAs), high-glucose levels, reactive oxidative species, and hypoxia (7). Once established, this inflammatory environment is thought to contribute to the development of metabolic disease symptoms, such as insulin resistance, caused by a disruption in normal metabolic signaling pathways.

In the adipose tissue of obese humans, B cells, together with IgG and IgM deposition, have been observed in crown-like structures (8), areas defined by accumulation of macrophages around dying adipocyte cells (9). Similarly, in wild-type mice, B cells are rarely observed within lean adipose tissue but begin to infiltrate after 4 weeks of mice being fed a high-fat diet (HFD) (10). Mice lacking both B and T cells do not show any differences in weight gain or induction of glucose intolerance on a HFD. Interestingly, however, obese adipose tissue has a greater macrophage infiltration in the absence of B and T cells, suggesting that lymphocytes may influence the recruitment of macrophages in obesity (10).

In humans, T2D is strongly linked to obesity (11), and many murine models of obesity have been used to study induction of T2D symptoms (12). Cytokine production by B cells is altered in both T2D patients and obese mice. In T2D patients, B cells constitutively secrete the pro-inflammatory chemoattractant IL-8, while also secreting lower levels of IL-10, TNFα, and IL-6 than non-diabetic controls (13). Similarly, LPS-stimulated B cells from obese mice secrete higher amounts of macrophage inflammatory protein-2 alpha (MIP-2), the murine ortholog of human IL-8, as well as lower levels of IL-10 (14). Thus, the pro-inflammatory phenotype seen in human T2D patients is recapitulated in obese mice. Not all cytokines behave in the same way, though, secretion of IL-6 and TNFα is increased in murine B cells from obese mice (14), yet decreased in human T2D patients (13). These differences might be species-specific or highlight the limitations of using obese murine models to model T2D. Overall, murine B cells will have a more pronounced inflammatory phenotype compared to the human counterpart in T2D.

## Lessons from B Cell-Deficient Mice

In order to define whether B cells are critical players in obesity and metabolic disease, different research groups used B cell-deficient mice, such as the μMT line, in which disruption of the B cell receptor μ chain, required for differentiation and survival of mature B cells, results in loss of B cells (15). Overall, μMT mice on a HFD developed obesity similarly to wild-type mice. Crucially, however, they did not present the glucose intolerance normally induced by HFD (14, 16). μMT mice had resting serum glucose and insulin levels closer to that of lean mice, as well as improved glucose and insulin tolerance test responses. This implies a strong role for B cells in promoting the development of T2D during obesity; nonetheless, the mechanism by which B cell exert this effect is still controversial.

One proposed mechanism is that B cells influence the development of T2D by promoting a pro-inflammatory phenotype in macrophages and T cells. μMT mice had fewer macrophages within the adipose tissue and increased expression of anti-inflammatory M2 markers, such as Arg 1 and Ym1 (14). Furthermore, B cell-deficient mice showed reduced serum levels of inflammatory cytokines such as TNFα, IL-6, and MCP-1, as well as reduced mRNA expression of IFNγ and CD8 within the adipose tissue (14). Thus, B cells appear to be involved in inducing a pro-inflammatory phenotype in both macrophages and T cells during obesity. Interestingly, μMT mouse adipocytes also secreted less leptin; however, the authors did not investigate if this is a direct or indirect effect of B cells (14).

How exactly B cells influence macro- phages and T cells has been in part addressed by another study, which also highlighted the importance of antibody secretion by B cells. Winer et al. (16) observed IgG antibodies within adipose tissue of obese mice and B cell infiltration of visceral adipose tissue (VAT) early in obesity development (16). Furthermore, the proportion of B cells within VAT that were class-switched (IgG+) increased over time when mice were fed a HFD. Splenic B cells from obese mice secreted more IgG and less IgM, which suggests that B cells are activated and switch to a memory phenotype during obesity. In the serum and adipose tissue, the IgG2c subclass, in particular, was significantly increased. Deposits of IgM and IgG were also reported in crown-like structures within VAT (16), similar to that observed in human (8). Winer et al. (16) proposed the obesity-induced antibody response to be pathogenic and to directly contribute to development of T2D via macrophages. Macrophages express Fc receptors that bind antibodies, the binding of which can be activating or inhibitory depending on the class of Fc receptor (17). Within the adipose tissue, μMT mice had fewer infiltrating M1-macrophages (16). Injection of purified IgG antibodies from obese mice in μMT mice increased the M1-macrophage infiltrate into VAT, as well as increasing macrophage TNFα secretion in culture (16). Most importantly, the symptoms of T2D were worsened in obese mice injected with purified IgG from obese mice, but not with IgG from lean mice (16). This strongly suggests the presence of pathogenic autoantibodies. How exactly the pathogenic IgG affects the phenotype of macrophages remains to be elucidated. It is important to note, however, that two additional publications reported lack of pathogenic IgGs (14) and no evidence of IgG secretion or class-switching of B cells (18), indicating that the involvement of antibodies in obesity may be model-dependent. Further studies with additional models are required to clarify this important point.

On a different level, Winer at al. (16) also observed less IFNγ+ CD8+ cells within obese adipose tissue in μMT. They proposed that, as well as influencing macrophages via immunoglobulin secretion; B cells also prime both CD4+ and CD8+ T cells via MHCI/II interaction. By specifically removing MHCI or MHCII from B cells, glucose tolerance and insulin sensitivity improved TNFα levels within the adipose tissue decreased and IFNγ secretion by CD8+ cells was down regulated (16). Importantly, the authors were then able to lessen the symptoms of obesity-dependent insulin resistance by depleting B cells with an anti-CD20 antibody. Although the depletion of B cells did not affect weight, the levels of IgM and IgG in the blood as well as the levels of TNFα and TNFα+ macrophages were reduced. As the depletion occurred after obesity was established, it suggests that B cells are required for both inducing and also maintaining the pro-inflammatory phenotype in macrophages and T cells that contributes to insulin resistance. It also indicates that targeting B cells therapeutically could improve the management of T2D.

## B Cells are also Involved in Adipose Tissue Homeostasis

In contrast to the pro-inflammatory role of B cells in promoting metabolic disease reported by others, Nishimura et al. (18) provided evidence of a protective role for a subset of B cells, regulatory B cells, also known as B10 or IL-10-producing B cells. IL-10 is an immunosuppressive cytokine strongly associated with reducing autoimmune disease and inflammation (19). Regulatory B cells or Bregs are a subset of B cells, which secrete IL-10 and TGF-β (19), mediating suppressive effects on other immune cells. In their research, Nishimura et al. (18) showed that murine B cells isolated from adipose tissue produce high levels of IL-10, even in the absence of any *in vitro* stimulation. In contrast, B cells isolated from the spleen and lymph nodes required stimulation in order to have detectable levels of IL-10.

To prove the hypothesis that Bregs influence adipose inflammation, B cell specific IL-10 knockout (KO) mice were established using bone marrow chimeras. To do this bone marrow cells comprising 10% IL-10 KO cells and 90% B cell KO cells were transferred into wild-type mice, such that all resulting B cells were IL-10 deficient (18). The infiltration of macrophages in adipose tissue increased, as well as the expression of pro-inflammatory markers such as CD44 and IFNγ (18). From these findings, it seems that there is a population of regulatory B cells within the adipose tissue that maintains homeostasis by suppressing pro-inflammatory responses, thus the production of IL-10 by B cells is important in counterbalancing insulin resistance.

The authors hypothesized that resident B cells within adipose tissue are dependent on local factors secreted by adipocytes, such as FFAs. Indeed, FFA supported adipose B cell survival and IL-10 production in culture (18). The binding of FFA to immune cells has previously been suggested to be via binding to toll-like receptor 4 (TLR4) (20). However, if FFA secretion helps support Bregs within adipose tissue, it does not explain their reduction in obesity. It is possible that the secretion of additional factors by obese adipocytes skew the Breg phenotype; however, this is not addressed in the paper.

## Concluding Remarks

**Figure 1:**
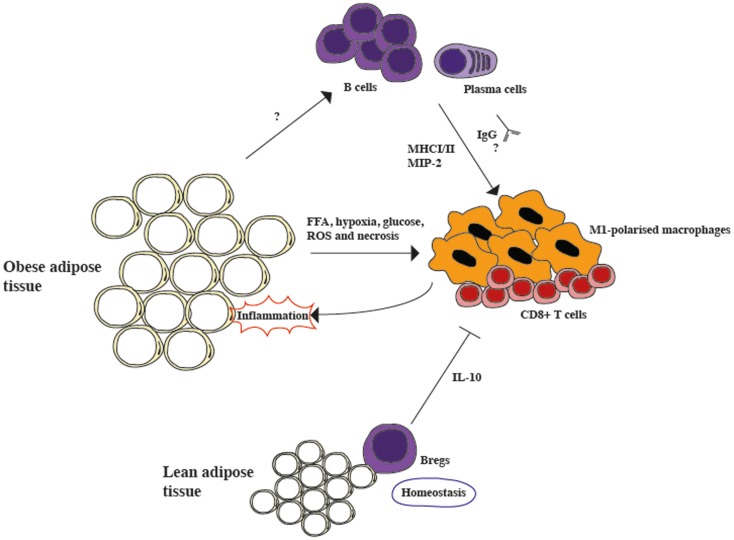
**The role of B cells in modulating inflammation in adipose tissue**. Lean adipose tissue contains resident Bregs, which constitutively secrete IL-10 and suppress inflammation during homeostasis. However, in obese adipose tissue the secretion of free fatty acids (FFA), hypoxia, high glucose levels, reactive oxygen species (ROS), and necrosis activates pro-inflammatory CD8+ cells and M1-polarized macrophages, generating inflammation. B cells are also activated during obesity, although the mechanism is still unclear. During obesity, B cells class-switch and secrete pathogenic IgGs, as well as the macrophage-recruiting chemokine, macrophage inflammatory protein-2 alpha (MIP-2). Furthermore, they also activate T cells via MHCI/II interactions. Overall, it seems that the protective role of Bregs is overcome by the pro-inflammatory role of activated B cells.

The idea that Bregs counterbalance the pro-inflammatory processes that characterize adipose tissue in obesity does not necessarily contrast with previous studies showing that the complete absence of B cells ameliorates inflammation and insulin resistance in obese mice. It is possible to speculate that Bregs are present in high numbers within the lean adipose tissue, possibly to avoid the development of inflammation in normal conditions. In support of this view, it has been reported that in humans IL-10 RNA levels in adipose tissue are inversely correlated with body mass index, whether this is due to Bregs or not remains to be established (18). Once homeostasis is lost, however, as observed in the adipose tissue of mice on HFD, the effect of activated B cells that prime T cells and generate immunoglobulins that affect macrophages, fueling the inflammatory response, overwhelms the protective effect of IL-10 producing Bregs (summarized in Figure 1). Experiments to determine how B cell subsets change during obesity progression and if individuals with lower levels of Bregs are more prone to insulin resistance would be required to further clarify the role of Bregs in obesity. Nonetheless, the observation that once obesity is established, insulin resistance can be ameliorated by B cell depletion via an anti-CD20 treatment offers an interesting therapeutic opportunity and warrants further investigation into B cell function in obesity.

## Further Questions for B Cells and Metabolism

In order to have a better understanding of how B cells affect all body-metabolism, it will also be necessary to gain more information on what metabolic pathways regulate B cells, their differentiation, cytokine production, and antibody secretion. Whereas glucose metabolism and the pentose phosphate pathways are necessary to support B cell clonal expansion (21), we still know little about the differentiation to antibody-secreting cells and cytokine production. Fatty acid biosynthesis is up regulated in antibody-secreting cells, in order to sustain the expansion of the endoplasmic reticulum to support antibody production (22). But what effect will an increase in free fatty acid uptake have on the B cell phenotype? Further research efforts are required to get a more comprehensive picture, which will ultimately help understanding the role of B cells in metabolic disorders and how to intervene therapeutically.

## Conflict of Interest Statement

The authors declare that the research was conducted in the absence of any commercial or financial relationships that could be construed as a potential conflict of interest.

## References

[1] Lund FE. Cytokine-producing B lymphocytes-key regulators of immunity. Curr Opin Immunol (2008) 20:332–8. 10.1016/j.coi.2008.03.003 18417336 PMC2474694

[2] Fain JN. Release of interleukins and other inflammatory cytokines by human adipose tissue is enhanced in obesity and primarily due to the nonfat cells. Vitam Horm (2006) 74:443–77. 10.1016/S0083-6729(06)74018-3 17027526

[3] Qatanani M Lazar MA. Mechanisms of obesity-associated insulin resistance: many choices on the menu. Genes Dev (2007) 21:1443–55. 10.1101/gad.1550907 17575046

[4] Agrawal S Gollapudi S Su H Gupta S. Leptin activates human B cells to secrete TNF-alpha, IL-6, and IL-10 via JAK2/STAT3 and p38MAPK/ERK1/2 signaling pathway. J Clin Immunol (2011) 31:472–8. 10.1007/s10875-010-9507-1 21243519 PMC3132280

[5] Lumeng CN Bodzin JL Saltiel AR. Obesity induces a phenotypic switch in adipose tissue macrophage polarization. J Clin Invest (2007) 117:175–84. 10.1172/JCI29881 17200717 PMC1716210

[6] Nishimura S Manabe I Nagasaki M Eto K Yamashita H Ohsugi M CD8+ effector T cells contribute to macrophage recruitment and adipose tissue inflammation in obesity. Nat Med (2009) 15:914–20. 10.1038/nm.1964 19633658

[7] Johnson AR Milner JJ Makowski L. The inflammation highway: metabolism accelerates inflammatory traffic in obesity. Immunol Rev (2012) 249:218–38. 10.1111/j.1600-065X.2012.01151.x 22889225 PMC3422768

[8] Mcdonnell ME Ganley-Leal LM Mehta A Bigornia SJ Mott M Rehman Q B lymphocytes in human subcutaneous adipose crown-like structures. Obesity (Silver Spring) (2012) 20:1372–8. 10.1038/oby.2012.54 22395812 PMC3682646

[9] Cinti S Mitchell G Barbatelli G Murano I Ceresi E Faloia E Adipocyte death defines macrophage localization and function in adipose tissue of obese mice and humans. J Lipid Res (2005) 46:2347–55. 10.1194/jlr.M500294-JLR200 16150820

[10] Duffaut C Galitzky J Lafontan M Bouloumie A. Unexpected trafficking of immune cells within the adipose tissue during the onset of obesity. Biochem Biophys Res Commun (2009) 384:482–5. 10.1016/j.bbrc.2009.05.002 19422792

[11] Colosia AD Palencia R Khan S. Prevalence of hypertension and obesity in patients with type 2 diabetes mellitus in observational studies: a systematic literature review. Diabetes Metab Syndr Obes (2013) 6:327–38. 10.2147/DMSO.S51325 24082791 PMC3785394

[12] Kanasaki K Koya D. Biology of obesity: lessons from animal models of obesity. J Biomed Biotechnol (2011) 2011:197636. 10.1155/2011/197636 21274264 PMC3022217

[13] Jagannathan M Mcdonnell M Liang Y Hasturk H Hetzel J Rubin D Toll-like receptors regulate B cell cytokine production in patients with diabetes. Diabetologia (2010) 53:1461–71. 10.1007/s00125-010-1730-z 20383694 PMC2895399

[14] Defuria J Belkina AC Jagannathan-Bogdan M Snyder-Cappione J Carr JD Nersesova YR B cells promote inflammation in obesity and type 2 diabetes through regulation of T-cell function and an inflammatory cytokine profile. Proc Natl Acad Sci U S A (2013) 110:5133–8. 10.1073/pnas.1215840110 23479618 PMC3612635

[15] Kitamura D Roes J Kuhn R Rajewsky K. A B cell-deficient mouse by targeted disruption of the membrane exon of the immunoglobulin mu chain gene. Nature (1991) 350:423–6. 10.1038/350423a0 1901381

[16] Winer DA Winer S Shen L Wadia PP Yantha J Paltser G B cells promote insulin resistance through modulation of T cells and production of pathogenic IgG antibodies. Nat Med (2011) 17:610–7. 10.1038/nm.2353 21499269 PMC3270885

[17] Nimmerjahn F Ravetch JV. Fcgamma receptors as regulators of immune responses. Nat Rev Immunol (2008) 8:34–47. 10.1038/nri2206 18064051

[18] Nishimura S Manabe I Takaki S Nagasaki M Otsu M Yamashita H Adipose natural regulatory B cells negatively control adipose tissue inflammation. Cell Metab (2013) 8:759–66. 10.1016/j.cmet.2013.09.017 24209772

[19] Mauri C Ehrenstein MR. The ‘short’ history of regulatory B cells. Trends Immunol (2008) 29:34–40. 10.1016/j.it.2007.10.004 18289504

[20] Eguchi K Manabe I Oishi-Tanaka Y Ohsugi M Kono N Ogata F Saturated fatty acid and TLR signaling link beta cell dysfunction and islet inflammation. Cell Metab (2012) 15:518–33. 10.1016/j.cmet.2012.01.023 22465073

[21] Doughty CA Bleiman BF Wagner DJ Dufort FJ Mataraza JM Roberts MF Antigen receptor-mediated changes in glucose metabolism in B lymphocytes: role of phosphatidylinositol 3-kinase signaling in the glycolytic control of growth. Blood (2006) 107:4458–65. 10.1182/blood-2005-12-4788 16449529 PMC1895797

[22] Fagone P Sriburi R Ward-Chapman C Frank M Wang J Gunter C Phospholipid biosynthesis program underlying membrane expansion during B-lymphocyte differentiation. J Biol Chem (2007) 282:7591–605. 10.1074/jbc.M608175200 17213195

